# Carga Inflamatória e Carga de Fibrilação Atrial: Uma Relação Bidirecional

**DOI:** 10.36660/abc.20230680

**Published:** 2024-07-01

**Authors:** Abdulrahman Naser, Samet Sayilan, Oya Güven, Büşra Güvendi Şengör, Atakan Biçici, Yücel Uzun, Ahmet Ekmekçi, Alev Kılıçgedik

**Affiliations:** 1 Kırklareli Training and Research Hospital Department of Cardiology Kırklareli Turquia Kırklareli Training and Research Hospital – Department of Cardiology, Kırklareli – Turquia; 2 Kırklareli University Medical School Kırklareli Training and Research Hospital Department of Internal Medicine Kırklareli Turquia Kırklareli University Medical School – Kırklareli Training and Research Hospital – Department of Internal Medicine, Kırklareli – Turquia; 3 Kırklareli University Medical School Kırklareli Training and Research Hospital Emergency Department Kırklareli Turquia Kırklareli University Medical School – Kırklareli Training and Research Hospital – Emergency Department, Kırklareli – Turquia; 4 Kartal Koşuyolu Training and Research Hospital Department of Cardiology İstanbul İstanbul Turquia Kartal Koşuyolu Training and Research Hospital – Department of Cardiology İstanbul, İstanbul – Turquia; 5 Bahçeşehir University Department of Cardiology İstanbul Turquia Bahçeşehir University – Department of Cardiology, İstanbul – Turquia; 6 Başakşehir Çam and Sakura City Hospital Department of Cardiology İstanbul Turquia Başakşehir Çam and Sakura City Hospital – Department of Cardiology, İstanbul – Turquia

**Keywords:** Inflamação, Fibrilação Atrial, Índice de Imunoinflamação Sistêmica

## Abstract

**Fundamento:**

A carga de fibrilação atrial (FA) é definida como a proporção de tempo que o paciente permanece em FA durante um determinado período de tempo; portanto, é teoricamente mais elevado na FA permanente e mais baixo na FA paroxística. A inflamação está associada ao início e à manutenção da FA. No entanto, a relação entre o índice de inflamação imune sistêmica (SII, do inglês *systemic immune-inflammation index*) e a carga de FA é desconhecida.

**Objetivo:**

No presente estudo, investigamos a relação entre o SII e a carga de FA.

**Métodos:**

O presente estudo é uma análise transversal de 453 pacientes (252 do sexo feminino e 201 do sexo masculino, com idade entre 44 e 94 anos) com FA (138 com FA paroxística e 315 com FA permanente) atendidos no ambulatório de cardiologia entre outubro de 2022 e junho de 2023. O SII foi calculado como (neutrófilos × plaquetas/linfócitos). O papel preditivo do SII e de outros marcadores inflamatórios na probabilidade do padrão de FA foi avaliado por análises de regressão logística, sendo considerado estatisticamente significativo o valor de p < 0,05.

**Resultados:**

Idade, pressão arterial diastólica, frequência cardíaca, diabetes mellitus, neutrófilos, relação plaquetas-linfócitos, relação neutrófilos-linfócitos, SII, proteína C reativa, largura de distribuição de glóbulos vermelhos, hemoglobina A1c e diâmetro do átrio esquerdo foram significativamente maiores no grupo com FA permanente. De acordo com a análise de regressão logística, idade (p = 0,038), diabetes mellitus (p = 0,024), largura de distribuição de glóbulos vermelhos (p = 0,023), proteína C reativa (p = 0,010), SII (p = 0,001) e o diâmetro do átrio esquerdo (p < 0,001) contribuíram significativamente para a predição da probabilidade de FA permanente.

**Conclusão:**

O SII está independentemente associado à carga de FA. Estudos prospectivos são necessários para determinar se o SII pode ser útil na identificação de pacientes com alto risco de progressão da FA.

## Graphical abstract

**Figura Central:**
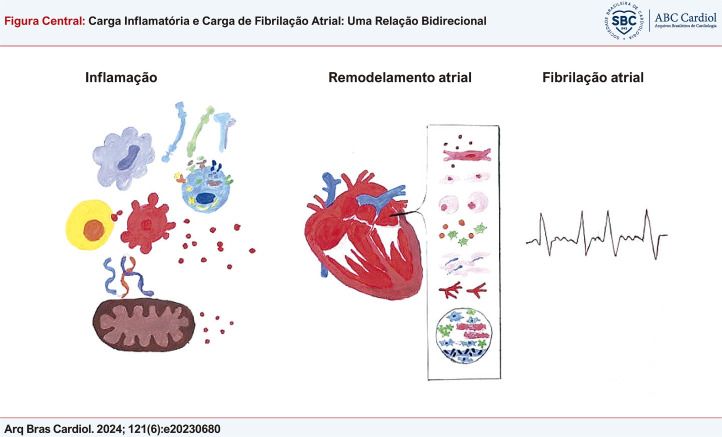
: Carga Inflamatória e Carga de Fibrilação Atrial: Uma Relação Bidirecional

*O SII está independentemente associado à carga de fibrilação atrial.*

## Introdução

A fibrilação atrial (FA) é uma arritmia cardíaca prevalente (2% a 4%) e grave que afeta mais de 33,5 milhões de indivíduos em todo o mundo. A FA pode causar acidente vascular cerebral e insuficiência cardíaca e aumenta a mortalidade e os custos de cuidados de saúde.^1,2^

A FA tem etiologias e mecanismos multifatoriais e intrincados. O envelhecimento, a genética, o diabetes, a hipertensão e a inflamação podem induzir alterações na estrutura atrial e na eletrofisiologia, e iniciar ou manter a FA.^1-3^ Esses fatores também facilitam a transição da FA da forma transitória (paroxística) para a forma persistente ou permanente.^2,3^ Anualmente, entre < 1% e 15% dos pacientes com FA paroxística evoluem para FA permanente.^3^ A carga de FA é definida como a proporção de tempo que um paciente permanece em FA durante um determinado período de monitoramento.^2^ É evidente que a carga de FA é maior em pacientes com FA permanente do que naqueles com FA paroxística. Maior carga de FA tem sido associada a resultados adversos, como insuficiência cardíaca, acidente vascular cerebral isquêmico e mortalidade.^4^Portanto, é essencial identificar e modificar os fatores de risco que podem promover a progressão da FA para sua forma permanente, a fim de prevenir complicações e melhorar o manejo e o prognóstico.

A contribuição exata da inflamação para o desenvolvimento da FA não está totalmente elucidada, mas é reconhecida como um fator-chave na patogênese da FA.^1^ A inflamação pode causar fibrose nos átrios, que é a principal característica do remodelamento estrutural.^1,5^ A inflamação também pode aumentar a incidência, a carga e a persistência da FA, bem como o risco tromboembólico relacionado à FA.^4,5^ Vários marcadores inflamatórios têm sido associados à FA, como proteína C reativa (PCR), contagem de leucócitos, plaquetas, fibrinogênio, fator de necrose tumoral-α, interleucinas e relação neutrófilos-linfócitos (RNL).^1,2,6,7^ A PCR é uma proteína de fase aguda que é o marcador inflamatório mais investigado na FA. Não está apenas associada à presença de FA, mas também pode prever o risco de desenvolver FA.^6^ Além disso, foi relatado que índices inflamatórios simples, como a RNL e a relação plaquetas-linfócitos (RPL), têm benefícios na previsão de FA.

O índice de inflamação imune sistêmica (SII, do inglês *systemic immune-inflammation index*) combina a RNL e a contagem de plaquetas. O SII reflete o estado inflamatório no sangue periférico e demonstrou prever doenças cardiovasculares, incluindo FA de início recente.^8^ No entanto, não foi adequadamente examinada a relação entre o SII e a carga de FA. Como a FA se sobrepõe em termos de classificação clínica e carga, no presente estudo, visamos investigar a associação entre dois estágios distintos de FA, paroxística e permanente (teoricamente aqueles com menor e maior carga de FA), e SII com uma visão mais simplificada.

## Métodos

Trata-se de um estudo transversal realizado entre outubro de 2022 e junho de 2023. Incluiu 453 pacientes (252 do sexo feminino e 201 do sexo masculino, com idade entre 44 e 94 anos) com FA (138 com FA paroxística e 315 com FA permanente) atendidos no ambulatório de cardiologia. Todos os pacientes foram acompanhados regularmente no ambulatório durante uma média de cinco anos e estavam sob terapia anticoagulante com anticoagulante oral não antagonista da vitamina K ou varfarina. Os critérios de inclusão do presente estudo foram ter FA paroxística ou permanente, concordar em participar do estudo e ter idade superior a 18 anos.

Os critérios de exclusão foram síndrome coronariana aguda, insuficiência renal aguda ou crônica grave, doença pulmonar obstrutiva crônica ou exacerbação de asma, apneia do sono, síndromes aórticas agudas, tromboembolismo venoso ou pulmonar agudo, doenças infecciosas, doenças da tireoide, distúrbios hematológicos, doenças inflamatórias ou reumatológicas crônicas, malignidades, FA pós-operatória, acidente vascular cerebral agudo, insuficiência cardíaca ou doença cardíaca valvar grave.

Foram registradas as características demográficas, pressão arterial, frequência cardíaca e ritmo do eletrocardiograma (ECG), dados ecocardiográficos e hábitos tabágicos dos pacientes. Foram obtidos hemograma completo, PCR e exames bioquímicos, como painel lipídico e função tireoidiana, hepática e renal. A RNL foi calculada como a razão entre as contagens de neutrófilos e linfócitos, e o SII foi calculado como o produto das contagens de neutrófilos e plaquetas dividido pela contagem de linfócitos. O presente estudo seguiu a Declaração de Helsinque e foi aprovado por um Comitê de Ética em Pesquisa local. O consentimento informado por escrito foi obtido de todos os participantes.

### Definição e diagnóstico de fibrilação atrial

Um ECG de 12 derivações foi realizado para todos os participantes e examinado por um cardiologista. A FA foi diagnosticada com base na presença de intervalos R-R irregulares, ausência de ondas P regulares e ativação atrial desorganizada no ECG. A FA foi classificada como paroxística se terminasse em 7 dias com ou sem intervenção. A FA foi classificada como permanente se fosse aceita pelo paciente e pelo médico, e nenhuma outra tentativa foi feita para restaurar ou manter o ritmo sinusal. No momento da inclusão no estudo, os ECGs basais dos pacientes com FA paroxística apresentavam ritmo sinusal, enquanto os ECGs basais dos pacientes com FA permanente mostravam ritmo de FA. Foram considerados ECGs pré-gravados e Holter de ritmo (para pacientes com aparelhos de Holter de ritmo registrado) no sistema hospitalar. As taxas de tratamento de ablação também foram registradas nos dados demográficos.

O estado dos sintomas foi caracterizado de acordo com a escala de sintomas da European Heart Rhythm Association (EHRA).^1^ Os pacientes cuja FA não causou nenhum sintoma foram definidos como assintomáticos (escore 1 da EHRA) e aqueles cuja FA causou palpitações, dispneia, fadiga ou outros sintomas relacionados à FA foram definidos como sintomáticos (EHRA 2 a 4).^1^

### Ecocardiografia

A ecocardiografia transtorácica foi realizada em todos os participantes. Os participantes foram avaliados em decúbito esquerdo, e foram registrados a fração de ejeção e o diâmetro anteroposterior do átrio esquerdo.

### Análise estatística

Foi usado o Statistical Package for the Social Sciences (SPSS) 17.0 (SPSS Inc., Chicago, EUA) para analisar os dados do presente estudo. Foi considerado estatisticamente significativo um valor de p < 0,05. O teste de Kolmogorov-Smirnov de uma amostra foi utilizado para verificar a normalidade dos dados. As variáveis contínuas com distribuição normal foram descritas como média e desvio padrão, e as variáveis contínuas sem distribuição normal foram descritas como mediana e intervalo interquartil (primeiro ao terceiro quartil). Os dados categóricos foram apresentados como números e porcentagens. Foram utilizados testes t de amostras independentes para comparar a diferença entre variáveis contínuas com distribuição normal, e o teste U de Mann-Whitney foi utilizado para comparar a diferença entre variáveis contínuas com distribuição não normal. O teste qui-quadrado e o teste exato de Fisher foram utilizados para avaliar a diferença entre as variáveis categóricas. A análise de regressão logística binária foi utilizada para identificar as variáveis independentes que contribuíram para a persistência da FA. Foram verificadas todas as premissas necessárias para a utilização da análise de regressão logística binária.

## Resultados

A Tabela 1 apresenta as características demográficas, antropométricas e clínicas da população do estudo. A média de idade dos pacientes com FA permanente foi significativamente maior que a dos pacientes com FA paroxística (73,44 ± 8,98 versus 70,32 ± 8,26, p = 0,001). Da mesma forma, as medianas da pressão arterial diastólica (80 [15] versus 80 [10], p = 0,041) e da frequência cardíaca (86 [24] versus 70 [16,25], p < 0,001) foram maiores e estatisticamente significativas em pacientes com FA permanente, comparados com pacientes com FA paroxística. Porém, não houve diferenças significativas entre os grupos em termos de sexo, antropometria, pressão arterial sistólica, , outras comorbidades além de diabetes mellitus (DM) e medicamentos. A frequência de DM foi significativamente maior em pacientes com FA permanente do que naqueles com FA paroxística (131 [41,6%] versus 36 [26,1%]). A FA assintomática também foi significativamente maior no grupo com FA permanente (p < 0,001). A taxa de tratamento de ablação foi significativamente maior em pacientes com FA paroxística (p = 0,011).

**Tabela 1:** – Características demográficas, antropométricas e clínicas da população estudada

Variáveis	FA paroxística 138	FA permanente 315	p
Sexo F/M n (%)	81/57 (54,3/45,7)	171/144 (58,7/41,3)	0,385
Idade (anos)	70,32±8,26	73,44±8,98	0,001
Altura (cm)	160 (155-169)	163 (158-170)	0,071
Peso (kg)	80 (72-90)	80 (74-92)	0,291
IMC	30,89 (26,67-35,18)	30,22 (27,64-34,05)	0,793
PAS (mmHg)	140 (125-140)	135 (120-145)	0,423
PAD (mmHg)	80 (70-80)	80 (70-85)	0,041
FC (bpm)	70 (63-79)	86 (74-98)	<0,001
Hipertensão n (%)	103 (74,6)	249 (79)	0,299
DM n (%)	36 (26,1)	131 (41,6)	0,002
DAC n (%)	26 (18,8)	75 (23,8)	0,242
Doença arterial periférica (%)	9 (6,5)	18 (5,7)	0,738
Tabagismo n (%)	14 (10,1)	18 (5,7)	0,090
Consumo de álcool n (%)	15 (10,9)	36 (11,4)	0,862
DPOC n (%)	8 (5,8)	21 (6,7)	0,728
Asma n (%)	6 (4,3)	10 (3,2)	0,583
Estado sintomático			
• Assintomático • Sintomático	58 (42) 80 (58)	221 (70,2) 94 (29,8)	<0,001
Tratamento de ablação			
• Recebeu • Não recebeu	26 (18,8) 112 (81,2)	32 (10,2) 283 (89,8)	0,011
NOAC n (%)	117 (84,8)	266 (84,4)	0,927
Varfarina n (%)	21 (15,2)	49 (15,6)	0,927
IECA/BRA n (%)	86 (62,3)	190 (60,3)	0,688
BB n (%)	78 (56,5)	195 (61,9)	0,281
BCC DHP n (%)	38 (28,4)	91 (30,4)	0,662
BCC não DHP n (%)	11 (8)	30 (9,5)	0,596
Estatina n (%)	34 (24,6)	54 (17,1)	0,063

*Os dados contínuos não paramétricos são apresentados como mediana (1º quartil a 3º quartil). BB: betabloqueadores; BCC: bloqueadores dos canais de cálcio; BRA: bloqueadores dos receptores da angiotensina; DAC: doença arterial coronariana; DHP: dihidropiridínicos; DM: diabetes mellitus; DPOC: doença pulmonar obstrutiva crônica; F: feminino; FA: fibrilação atrial; FC: frequência cardíaca; IECA: inibidores da enzima conversora de angiotensina; IMC: índice de massa corporal; M: masculino; NOAC: anticoagulante oral não antagonista da vitamina K; PAD: pressão arterial diastólica; PAS: pressão arterial sistólica.*

Marcadores inflamatórios convencionais, como contagem de neutrófilos, RNL, RPL, SII, PCR e largura de distribuição dos glóbulos vermelhos (RDW, do inglês *red blood cell distribution width*) foram significativamente maiores em pacientes com FA permanente do que em pacientes com FA paroxística. Pelo contrário, a contagem média de linfócitos dos pacientes com FA permanente foi significativamente menor do que a dos pacientes com FA paroxística. No entanto, a contagem de leucócitos, hemoglobina e contagem de plaquetas não diferiu significativamente entre os grupos, conforme mostrado na Tabela 2. Da mesma forma, os testes de função renal, hepática e tireoidiana, bem como os resultados do painel lipídico e da glicemia em jejum não diferiram significativamente entre os dois grupos. Em contraste, a hemoglobina A1C foi significativamente maior em pacientes com FA permanente do que em pacientes com FA paroxística. Em relação aos dados ecocardiográficos, a fração de ejeção de ambos os grupos foi semelhante, mas o diâmetro do átrio esquerdo dos pacientes com FA permanente foi significativamente maior que o dos pacientes com FA paroxística.

**Tabela 2:** – Características laboratoriais e ecocardiográficas da população estudada

Variáveis	FA paroxística 138	FA permanente 315	p
WBC (×10^3^/µL)	7,04±1,78	7,28±1,91	0,218
Hemoglobina (g/dL)	13,26±1,69	12,93±1,84	0,070
Neutrófilos (×10^3^/µL)	3,875 (2,958-5,000)	4,400 (3,480-5,770)	<0,001
Linfócitos (×10^3^/µL)	2,15±0,67	1,82±0,7	<0,001
Plaquetas (×10^3^/µL)	229,5 (191,0-288,5)	229 (198-272)	0,743
RPL	109,75 (90,46-139,90)	133,94 (98,15-180,95)	<0,001
RNL	1,86 (1,36-2,57)	2,39 (1,85-3,47)	<0,001
SII	444,11 (309,25-601,12)	562,50 (386,41-897,66)	<0,001
PCR (mg/L)	2,42 (1,58-5,10)	4,60 (2,20-11,10)	<0,001
RDW (%)	14,5 (13,7-15,3)	15,0 (14,2-16,3)	<0,001
Creatinina (mg/dL)	0,84±0,15	0,86±0,17	0,246
TFG (ml/minute)	83,27 (68,44-99,45)	75,76 (65,95-93,54)	0,051
ALT (U/L)	15 (11-21)	15 (11-20)	0,989
AST (U/L)	18 (16-23)	19 (15-23)	0,959
TSH (ng/dL)	2,19 (1,08-3,15)	2,2 (1,41-3,15)	0,278
T3 (ng/dL)	3,8 (2,7-4,6)	4 (3,07-4,60)	0,171
T4 (ng/dL)	16,45 (14,70-18,20)	16,20 14,40-18,20)	0,565
GJ (mg/dL)	109 (98-131)	114 (98-145)	0,111
HBA1C (%)	5,8 (5,5-6,3)	5,9 (5,54-6,90)	0,047
CT (mg/dL)	188,55±43,04	180,60±41,46	0,065
LDL-C (mg/dL)	109 (81-134)	107 (82-128)	0,344
HDL-C (mg/dL)	47 (39-56)	46 (39-53)	0,080
TG (mg/dL)	127 (99-183)	124 (91-165)	0,173
FE (%)	65 (60-65)	65 (60-65)	0,503
DAE (cm)	38,6 (38,0-41,0)	46 (43-49)	<0,001

*Os dados contínuos não paramétricos são apresentados como mediana (1º quartil a 3º quartil). ALT: alanina aminotransferase; AST: aspartato aminotransferase; CT: colesterol total; DAE: diâmetro do átrio esquerdo; FA: fibrilação atrial; FE: fração de ejeção; GJ: glicemia de jejum; HBA1C: hemoglobina A1C; HDL-C: colesterol de lipoproteína de alta densidade; LDL-C: colesterol de lipoproteína de baixa densidade; PCR: proteína C reativa; RDW: largura de distribuição dos glóbulos vermelhos; RNL: relação neutrófilos/linfócitos; RPL: relação plaquetas/linfócitos; SII: índice de inflamação imune sistêmica; TFG: taxa de filtração glomerular; TG: triglicerídeo; TSH: hormônio estimulador da tireoide; T3: triiodotironina; T4: tetraiodotironina; WBC: contagem de leucócitos.*

Foi realizada análise de regressão logística binária para identificar as variáveis independentes que influenciaram a permanência da FA. Para tanto, foi examinado o efeito da idade, sexo, índice de massa corporal (IMC), hipertensão, doença arterial coronariana, DM, doença pulmonar obstrutiva crônica, leucócitos, RDW, PCR, SII e diâmetro anteroposterior do átrio esquerdo no padrão de FA. O modelo completo com todas as variáveis citadas foi estatisticamente significativo (X^2^ [12, n = 453] = 324,20, p < 0,001), indicando que o modelo foi capaz de distinguir o padrão de FA como paroxístico ou permanente. Nosso modelo ajustou bem os dados (bom ajuste) com um valor de significância no teste de Hosmer e Lemeshow de p = 0,947. O modelo também apresentou alto percentual (88,5%) de precisão de predição. Além disso, de 51,1% (R quadrado de Cox e Snell) a 72,2% (R quadrado de Nagelkerke) da variância no padrão de AF podia ser explicada pelo modelo. Conforme a Tabela 3, as variáveis idade, DM, leucócitos, RDW, PCR e SII estiveram significativamente associadas ao padrão de FA. Considerando a idade, as chances de ter FA permanente aumentaram 1.046 vezes a cada ano de aumento de idade. Da mesma forma, indivíduos com DM tiveram chances 2,2 vezes maiores de ter FA permanente do que pacientes sem DM. O RDW teve uma razão de chances (OR) de 1,31, o que significa que as chances de ter FA permanente aumentaram 1,31 vezes por unidade de aumento no RDW. Da mesma forma, um aumento unitário na PCR aumentou as chances de ter FA permanente em 1,31 vezes. O SII foi outro preditor significativo de FA permanente (OR: 1,002, p = 0,001). Isso significa que para cada aumento unitário no SII, a probabilidade de ter FA permanente mudou por um fator de 1,002. O diâmetro anteroposterior do átrio esquerdo foi detectado como a variável mais fortemente associada à FA permanente neste modelo, com um OR de 2,04, indicando que cada centímetro de aumento no diâmetro do átrio esquerdo aumentou as chances de ter FA permanente em 2,04 vezes, conforme mostrado na Tabela 3.

**Tabela 3:** – Análise de regressão logística binária para a probabilidade do padrão de fibrilação atrial

Variáveis	B	SE	Wald	df	p	OR	IC 95% da OR	
							Inferior	Superior
Idade	0,045	0,022	4,288	1	0,038	1,046	1,002	1,092
Sexo (feminino)	−0,471	0,372	1,608	1	0,205	0,624	0,301	1,293
IMC	−0,012	0,033	0,134	1	0,714	0,988	0,926	1,054
HT (presente)	−0,304	0,412	0,545	1	0,460	0,738	0,329	1,654
DAC (presente)	−0,085	0,441	0,037	1	0,847	0,919	0,387	2,179
DM (presente)	0,789	0,387	4,153	1	0,042	2,201	1,031	4,698
DPOC (presente)	0,350	0,751	0,218	1	0,641	1,420	0,326	6,181
WBC	−0,084	0,110	0,581	1	0,446	0,919	0,741	1,141
RDW	0,270	0,119	5,137	1	0,023	1,310	1,037	1,654
PCR	0,108	0,042	6,595	1	0,010	1,114	1,026	1,210
SII	0,002	0,001	10,414	1	0,001	1,002	1,001	1,003
DAE	0,713	0,076	87,486	1	<0,001	2,040	1,757	2,369
Constante	−36,644	4,555	64,726	1	<0,001	0,000		

*DAC: doença arterial coronariana; DAE: diâmetro do átrio esquerdo; df: graus de liberdade; DM: diabetes mellitus; DPOC: doença pulmonar obstrutiva crônica; HT: hipertensão; IC: intervalo de confiança; IMC: índice de massa corporal; OR: razão de chances; PCR: proteína C reativa; RDW: largura de distribuição dos glóbulos vermelhos; SE: erro padrão; SII: índice de inflamação imune sistêmica; WBC: contagem de leucócitos.*

### Pacientes com diabetes

A Tabela Suplementar 1 descreve algumas características dos pacientes com diabetes. Um total de 167 (36,9%) dos 453 pacientes tinham diabetes. A mediana do IMC dos pacientes com DM foi de 30,81 (27,64 a 34,68), e eles tinham diabetes há uma mediana de 10 (6 a 13) anos. Destes 167 pacientes, 147 (88%) estavam em uso de antidiabéticos orais e 40 (24%) em terapia com insulina. A maioria (n = 131, 78,4%) destes pacientes apresentava FA permanente. O RDW como marcador inflamatório foi significativamente maior em pacientes com DM em comparação com aqueles sem DM.

### Tratamento de ablação

Na presente amostra, 58 (12,8%) pacientes foram submetidos à ablação. Os pacientes submetidos à ablação eram significativamente mais jovens, mais frequentemente do sexo masculino e tinham IMC mais baixo. Porém, a frequência de tabagismo foi maior nos pacientes submetidos à ablação (Tabela Suplementar 2).

### Estado sintomático/assintomático dos pacientes

Embora a maioria dos pacientes com FA paroxística fossem significativamente sintomáticos (80/138), a maioria dos pacientes com FA permanente eram assintomáticos (221/315). O IMC, a frequência de DM, doença arterial coronariana e doença pulmonar obstrutiva crônica foram significativamente maiores nos pacientes sintomáticos. No grupo assintomático, o uso de betabloqueadores foi significativamente maior (Tabela Suplementar 3).

## Discussão

O objetivo do presente estudo foi investigar a relação entre inflamação e carga de FA. Verificamos que pacientes com FA permanente apresentavam maior carga inflamatória do que pacientes com FA paroxística. Isso foi evidenciado por níveis significativamente mais elevados de neutrófilos, RPL, RNL, SII, PCR e RDW em pacientes com FA permanente. Além disso, idade, diabetes, RDW, PCR, SII (ilustração central) e diâmetro anteroposterior do átrio esquerdo foram independentemente associados ao padrão de FA permanente, que apresenta a carga qualitativamente mais alta de FA.

Acredita-se que a inflamação desempenhe um papel fundamental na patogênese da FA. Foi demonstrado que os marcadores inflamatórios estão elevados em pacientes com FA, e a carga inflamatória demonstrou estar associada à carga e ao prognóstico da FA. Os mecanismos exatos pelos quais a inflamação contribui para a FA não são totalmente compreendidos, mas podem envolver remodelação elétrica e estrutural dos átrios.^1,5^

A prevalência de FA aumenta com a idade em ambos os sexos, duplicando a cada 10 anos e atingindo até 20% em pessoas com mais de 80 anos.^1,9,10^ A idade avançada é também um preditor independente de FA permanente, como demonstrado por um estudo com acompanhamento de 30 anos que relatou uma probabilidade cumulativa de 29% de FA não permanente progredir para FA permanente.^1,11^ Nossos achados são consistentes com estudos anteriores que demonstraram uma associação positiva entre idade e progressão da FA.^10-12^ Os possíveis mecanismos subjacentes a essa associação incluem a maior incidência de comorbidades, inflamação e fibrose atrial na população idosa.^13^

O DM é um fator de risco bem estabelecido para o desenvolvimento de FA, com risco 34% maior em comparação com indivíduos sem DM.^4,14^ No entanto, o papel do diabetes na manutenção e progressão da FA não é totalmente compreendido, e os resultados de estudos anteriores são conflitantes.^1,15,16^ No presente estudo, a FA permanente foi significativamente maior em pacientes com DM do que naqueles sem DM. Além disso, a RDW como marcador inflamatório foi significativamente maior em pacientes com DM em comparação com aqueles sem DM. Em nosso estudo, verificamos uma associação positiva e independente entre diabetes e FA permanente. Apesar do desenho diferente, das características populacionais e do tamanho amostral menor em comparação aos estudos anteriores, resultados semelhantes foram obtidos entre DM e FA na presente análise. Essa relação pode ser explicada pela elevada carga inflamatória no DM.

O aumento do átrio esquerdo é consequência do aumento da pressão e do volume atrial, bem como da disfunção diastólica do ventrículo esquerdo, que levam ao remodelamento elétrico e estrutural do átrio esquerdo e predispõe ao início e à progressão da FA.^1,4^ Por outro lado, a FA causa maior dilatação atrial, criando um ciclo vicioso. Um estudo recente de Menichelli et al.^17^ relatou um diâmetro mediano do átrio esquerdo significativamente maior em pacientes com FA permanente do que naqueles com FA paroxística (≥ 44 mm, 59,5% versus 37,5% respectivamente, p < 0,001). Além disso, a FA permanente e o aumento do átrio esquerdo estão associados a um maior risco de acidente vascular cerebral isquêmico e embolia sistémica.^4,17,18^Um estudo recente também encontrou um grau significativamente mais elevado de remodelamento do átrio esquerdo em termos de tamanho mais aumentado e função mais prejudicada (átrio esquerdo rígido) em pacientes com FA permanente em comparação com FA paroxística, refletindo diferentes estágios da doença.^19^ Nesse contexto, nossa análise mostrou que o diâmetro anteroposterior do átrio esquerdo (uma medida simplificada do tamanho do átrio esquerdo) estava associado a FA permanente.

A inflamação é considerada um potencial gatilho e um fator perpetuador na fisiopatologia da FA.^5^ Vários estudos de caso-controle demonstraram níveis mais elevados de marcadores inflamatórios em pacientes com FA em comparação com aqueles sem FA.^5,20-22^ Além disso, a carga inflamatória e a fibrose atrial estão positivamente correlacionadas com a carga de FA.^5,23^ Smit et al.^24^ relataram que marcadores inflamatórios estavam associados ao desenvolvimento de FA permanente. Em um grande estudo de base populacional, Aviles et al. relataram que o aumento da PCR estava associado à presença de FA e à previsão de FA futura.^6^ De forma semelhante, nosso estudo verificou que a FA permanente estava associada a níveis mais elevados de PCR do que a FA paroxística, sugerindo que os níveis de PCR podem refletir a carga de FA.^25^ O RDW é outro indicador importante de inflamação e tem sido associado à ocorrência, recorrência, permanência da FA e a eventos adversos relacionados à FA.^26^ Além disso, o RDW demonstrou ser um preditor independente de FA pós-operatória após cirurgia de revascularização do miocárdio.^27^ Além disso, o RDW elevado tem sido sugerido como um preditor independente de desfechos clínicos adversos a longo prazo.^26,28^ Wan et al. relataram que indivíduos com níveis elevados de RDW exibiram uma prevalência significativamente maior de FA persistente e permanente.^28^ Além disso, o RDW foi independentemente associado à progressão da FA de paroxística para permanente.^29^ Nossos resultados apoiam a literatura prévia ao observar uma associação independente entre valores de RDW e FA permanente. Em resumo, nossos achados em relação aos marcadores inflamatórios como RDW, PCR e SII mostram que eles estão associados à carga e à permanência da FA.

O SII é um novo marcador inflamatório que combina contagens de plaquetas, neutrófilos e linfócitos e pode refletir o estado imunológico e inflamatório com mais precisão do que qualquer uma dessas células isoladamente. Foi demonstrado que o SII elevado está significativamente associado a vários eventos e desfechos cardiovasculares.^9,30-32^ Além disso, o SII foi proposto como um preditor de desenvolvimento de FA após cirurgia de revascularização do miocárdio,^33^ recorrência de FA após procedimento de crioablação com cirurgia da valva mitral^31,34^ e após cardioversão por corrente direta bem-sucedida.^35^O presente estudo tentou explorar a associação entre a carga de FA, que é teórica e qualitativamente mais alta na FA permanente e mais baixa na FA paroxística, e SII, em um método específico e com uma visão mais simplificada.

Considerando que a inflamação está intimamente relacionada ao desenvolvimento, carga e progressão da FA, possíveis medidas preventivas podem prevenir todas essas etapas.^6,36^ Adicionalmente, agentes direcionados a biomarcadores inflamatórios recentemente começaram a ser investigados como potenciais medicamentos no tratamento da FA.^6,36,37^ Além disso, o tratamento com colchicina após isolamento das veias pulmonares para FA paroxística está associado a menor taxa de recorrência de FA.^34^ Isso indica que a redução desses marcadores pró-inflamatórios poderia orientar a escolha dos melhores perfis de pacientes para resposta ao tratamento clínico ou intervenção com cateter.

A ablação por cateter é cada vez mais utilizada para controle do ritmo no tratamento da FA.^1^ Sabe-se que as características demográficas têm um impacto significativo na resposta ao tratamento e nos resultados da ablação por cateter.^1,38^ Em um estudo realizado por Kummer et al., os pacientes submetidos a ablação por cateter eram significativamente mais jovens, mais frequentemente do sexo masculino, mais frequentemente brancos e mais frequentemente segurados de forma privada, com rendimentos familiares mais elevados e taxas mais baixas de comorbilidade médica.^39^ Na presente amostra, todos os pacientes estavam segurados através de programas de seguros patrocinados pelo governo e 12,8% (58/453) dos pacientes foram submetidos à ablação. Os pacientes submetidos à ablação com maior frequência apresentavam FA paroxística, IMC mais baixo.

Embora a FA seja frequentemente assintomática, ela pode ser incapacitante.^1^ O tipo de FA e a presença de comorbidades são fatores eficazes para determinar se os pacientes com FA serão sintomáticos ou não.^1,40^ Foi relatado que a FA permanente é 3 vezes mais comum em pacientes assintomáticos do que em pacientes sintomáticos.^40^ Além disso, foi demonstrado que sexo masculino, idade avançada, infarto do miocárdio prévio e atividade física limitada estão significativamente associados à FA assintomática.^40^ Consistente com a literatura prévia, a frequência de FA paroxística em pacientes sintomáticos e a frequência de FA permanente em pacientes assintomáticos foram significativamente maiores. Da mesma forma, as comorbidades foram significativamente maiores no grupo sintomático.

### Limitações

Em primeiro lugar, a carga de FA é um conceito complexo que não pode ser medido com precisão. Na presente análise, a carga de FA foi estimada aproximadamente a partir dos padrões de FA, sendo qualitativamente mais baixa e mais alta na FA paroxística e permanente, respectivamente. Em segundo lugar, trata-se de um estudo transversal unicêntrico; portanto, não pode provar causalidade. Estudos prospectivos com acompanhamento de longo prazo são necessários para validar nossos achados e investigar os mecanismos subjacentes da inflamação na FA. Em terceiro lugar, as classificações clínicas atualmente utilizadas na FA refletem inadequadamente a persistência temporal da FA. Além disso, os pacientes classificados na mesma classe clínica de FA podem ser inerentemente heterogêneos em termos de persistência temporal de FA.^41^ Visamos explorar a relação entre o SII e dois estágios distintos de FA, paroxística e permanente (teoricamente aqueles com menor e menor maior carga de AF). Portanto, o estudo não incluiu um grupo controle sem FA, mas com as mesmas características demográficas, nem um grupo com as formas persistente e persistente de longa duração de FA. No entanto, seria mais valioso revelar a relação gradual entre o aumento da carga de FA (presumivelmente de acordo com a temporalidade da FA, de paroxística a persistente, persistente de longa duração e permanente) e SII. Portanto, são necessários estudos prospectivos bem desenhados para darem sentido à nossa pesquisa. Em quarto lugar, a população do nosso estudo era relativamente pequena e consistia de pacientes idosos e caucasianos; portanto, a generalização de nossos achados para outras populações é incerta. Em quinto lugar, utilizamos apenas o diâmetro anteroposterior do átrio esquerdo, um parâmetro simples que não reflete verdadeiramente o tamanho do átrio esquerdo, principalmente no caso de átrio esquerdo assimétrico. Portanto, estudos que avaliem o volume atrial esquerdo para determinar o tamanho do átrio esquerdo contribuirão para nossa análise.

## Conclusão

Além do aumento do átrio esquerdo, a carga inflamatória representada por SII, RNL, PCR e RDW está independentemente associada à carga de FA. Nosso estudo fornece informações importantes sobre a relação entre inflamação e carga de FA. No entanto, são necessárias mais pesquisas para validar os nossos achados e investigar o papel potencial de terapias anti-inflamatórias na prevenção ou no tratamento da FA.
