# Supplementary material for: Carga Inflamatória e Carga de Fibrilação Atrial: Uma Relação Bidirecional
Source: Arq Bras Cardiol. 2024 Jul 1;121(6):e20230680. [Article in Portuguese] doi: 10.36660/abc.20230680 (PMC12080626; doi:10.36660/abc.20230680)
Supplement: Translation

# Translation: Inflammation Burden and Atrial Fibrillation Burden: A Bidirectional Relationship

**DOI:** 10.36660/abc.20230680i

**Authors:** Abdulrahman Naser, Samet Sayilan, Oya Güven, Büşra Güvendi Şengör, Atakan Biçici, Yücel Uzun, Ahmet Ekmekçi, Alev Kılıçgedik

**Affiliations:** 1 Turkey Kırklareli Training and Research Hospital – Department of Cardiology, Kırklareli – Turkey; 2 Turkey Kırklareli University Medical School – Kırklareli Training and Research Hospital – Department of Internal Medicine, Kırklareli – Turkey; 3 Turkey Kırklareli University Medical School – Kırklareli Training and Research Hospital – Emergency Department, Kırklareli – Turkey; 4 Turkey Kartal Koşuyolu Training and Research Hospital – Department of Cardiology İstanbul, İstanbul – Turkey; 5 Turkey Bahçeşehir University – Department of Cardiology, İstanbul – Turkey; 6 Turkey Başakşehir Çam and Sakura City Hospital – Department of Cardiology, İstanbul – Turkey

**Keywords:** Inflammation, Atrial Fibrillation, Systemic Immune-inflammation Index

## Abstract

**Background:**

Atrial fibrillation (AF) burden is defined as the proportion of time the patient remains in AF over a given period of time; thus, it is theoretically highest in permanent AF and lowest in paroxysmal AF. Inflammation is associated with the initiation and maintenance of AF. However, the relationship between systemic immune-inflammation index (SII) and AF burden is unknown.

**Objective:**

In the present study, we investigated the relationship between SII and AF burden.

**Methods:**

The present study is a cross-sectional analysis of 453 patients (252 females and 201 males, aged 44 to 94 years) with AF (138 with paroxysmal AF and 315 with permanent AF) who visited the cardiology outpatient clinic between October 2022 and June 2023. SII was calculated as (neutrophils × platelets/lymphocytes). The predictive role of SII and other inflammatory markers in the likelihood of AF pattern was evaluated by logistic regression analyses, and p value < 0.05 was considered statistically significant.

**Results:**

Age, diastolic blood pressure, heart rate, diabetes mellitus, neutrophil, platelet-to-lymphocyte ratio, neutrophil-to-lymphocyte ratio, SII, C-reactive protein, red blood cell distribution width, hemoglobin A1c, and left atrial diameter were significantly higher in the permanent AF group. According to the logistic regression analysis, age (p = 0.038), diabetes mellitus (p = 0.024), red blood cell distribution width (p = 0.023), C-reactive protein (p = 0.010), SII (p = 0.001), and left atrial diameter (p < 0.001) significantly contributed to the prediction of the likelihood of permanent AF.

**Conclusion:**

SII is independently associated with the AF burden. Prospective studies are needed to determine whether SII may be useful in identifying patients at high risk for AF progression.

## Introduction

Atrial fibrillation (AF) is a prevalent (2% to 4%) and severe cardiac arrhythmia that affects over 33.5 million individuals globally. AF can lead to stroke and heart failure, and it elevates mortality and healthcare costs.^1,2^

AF has multifactorial and intricate etiologies and mechanisms. Aging, genetics, diabetes, hypertension, and inflammation can induce alterations in the atrial structure and electrophysiology and initiate or sustain AF.^1-3^ These factors also facilitate the transition of AF from transient (paroxysmal) to persistent or permanent forms.^2,3^ Annually, < 1% to 15% of patients with paroxysmal AF progress to permanent AF.^3^ AF burden is defined as the proportion of time that a patient is in AF during a certain monitoring period.^2^ It is evident that AF burden is higher in patients with permanent AF than in those with paroxysmal AF. Higher AF burden has been associated with adverse outcomes such as heart failure, ischemic stroke, and mortality.^4^Therefore, it is essential to identify and modify the risk factors that can promote the progression of AF to its permanent form, in order to prevent complications and enhance management and prognosis.

The exact contribution of inflammation to the development of AF is not fully elucidated, but it is recognized as a key factor in the pathogenesis of AF.^1^ Inflammation can cause fibrosis in the atria, which is the main characteristic of structural remodeling.^1,5^ Inflammation can also augment the incidence, burden, and persistence of AF, as well as the thromboembolic risk related to AF.^4,5^ Several inflammatory markers have been associated with AF, such as C-reactive protein (CRP), white blood cell (WBC), platelet, fibrinogen, tumor necrosis factor-α, interleukins, and neutrophil-to-lymphocyte ratio (NLR).^1,2,6,7^ CRP is an acute phase protein that is the most investigated inflammatory marker in AF. It is not only associated with the presence of AF, but also can forecast the risk of developing AF.^6^ Furthermore, simple inflammatory indices such as NLR and platelet-to-lymphocyte ratio (PLR) have been reported to be beneficial in predicting AF.

The systemic immune-inflammation index (SII) is a combination of NLR and platelet count. It reflects the inflammation status in peripheral blood and has been demonstrated to predict cardiovascular disease including new onset AF.^8^ However, the relationship between SII and AF burden has not been adequately examined. As AF overlaps in terms of clinical classification and burden, in this study, we aimed to investigate the association between two distinctly separate stages of AF, paroxysmal and permanent (theoretically those with the lowest and highest AF burden), and SII with a more simplified view.

## Methods

This study was a cross-sectional study conducted between October 2022 and June 2023. It included 453 patients (252 females and 201 males, aged 44 to 94 years) with AF (138 with paroxysmal AF and 315 with permanent AF) who visited the cardiology outpatient clinic. All patients were regularly followed up at the outpatient clinic for a median of five year, and were under anticoagulant therapy with either a non-vitamin K antagonist oral anticoagulant or warfarin. The inclusion criteria for this study were having paroxysmal or permanent AF, agreeing to participate in this study, and being older than 18 years of age.

The exclusion criteria were acute coronary syndrome, acute or severe chronic renal failure, chronic obstructive pulmonary disease or asthma exacerbation, sleep apnea, acute aortic syndromes, acute venous or pulmonary thromboembolism, infectious disease, thyroid disease, hematologic disorders, chronic inflammatory or rheumatologic disease, malignancies, postoperative AF, acute stroke, heart failure, or severe valvular heart disease.

The demographic characteristics, blood pressure, electrocardiogram (ECG) rhythm and heart rate, echocardiographic data, and smoking habits of the patients were recorded. Complete blood count, CRP, and biochemical tests, such as lipid panel and thyroid, liver and kidney functions, were performed. NLR was calculated as the ratio of neutrophil to lymphocyte counts, and SII was calculated as the product of neutrophil and platelet counts divided by the lymphocyte count. This study followed the Helsinki Declaration and was approved by a local Research Ethics Committee. Written informed consent was obtained from all participants.

### Definition and diagnosis of atrial fibrillation

A 12-lead ECG was performed for all participants and examined by a cardiologist. AF was diagnosed based on the presence of irregularly irregular R-R intervals, absence of regular P waves, and disorganized atrial activations on the ECG. AF was classified as permanent if it was accepted by the patient and physician, and no further attempts were made to restore or maintain sinus rhythm. At the time of enrollment in the study, the baseline ECGs of the patients with paroxysmal AF showed sinus rhythm, while the baseline ECGs of the patients with permanent AF showed AF rhythm. Pre-recorded ECGs and rhythm Holter (for patients with recorded rhythm Holter devices) in the hospital system were taken into account. Rates of ablation treatment were also recorded in demographic data.

Symptom status was characterized according to the European Heart Rhythm Association (EHRA) symptom scale.^1^ Patients whose AF did not cause any symptoms were defined as asymptomatic (EHRA score 1), and those whose AF caused palpitations, dyspnea, fatigue, or other AF-related symptoms were defined as symptomatic (EHRA 2 to 4).^1^

### Echocardiography

Transthoracic echocardiography was performed for all participants. Participants were evaluated in the left decubitus position, and their ejection fraction and left atrial anterior-posterior diameter were recorded.

### Statistical analysis

Statistical Package for the Social Sciences (SPSS) 17.0 (SPSS Inc., Chicago, USA) was used to analyze the data of the present study. A p value of < 0.05 was considered statistically significant. One sample Kolmogorov-Smirnov test was used to verify the normality of the data. Normally distributed continuous variables were described as mean and standard deviation, and continuous variables without normal distribution were described as median and interquartile range (first to third quartile). Categorical data were presented as numbers and percentages. Independent samples t tests were used to compare the difference between normally distributed continuous variables, and the Mann-Whitney U test was used to compare the difference between non-normally distributed continuous variables. The chi-square test and Fisher’s exact test were used to evaluate the difference between categorical variables. Binary logistic regression analysis was used to identify the independent variables contributing to the persistence of AF. All the necessary assumptions were verified for the use of binary logistic regression analysis.

## Results

Table 1 shows the demographic, anthropometric, and clinical characteristics of the study population. The mean age of individuals with permanent AF was significantly higher than individuals with paroxysmal AF (73.44 ± 8.98 versus 70.32 ± 8.26, p = 0.001). Similarly, the median diastolic blood pressure (80 [15] versus 80 [10], p = 0.041) and heart rate (86 [24] versus 70 [16.25], p < 0.001) were higher and statistically significant in patients with permanent AF compared to patients with paroxysmal AF. However, there were no significant differences between groups in terms of sex, anthropometrics, systolic blood pressure, comorbidities other than diabetes mellitus (DM), and medications. The frequency of DM was significantly higher in patients with permanent AF than paroxysmal AF (131 [41.6%] versus 36 [26.1%]). Asymptomatic AF was also significantly higher in the permanent AF group (p < 0.001). The rate of ablation treatment was significantly higher in patients with paroxysmal AF (p = 0.011).

**Table 1:** – Demographic, anthropometric, and clinical characteristics of the study population

Variables	Paroxysmal AF 138	Permanent AF 315	p
Sex F/M n (%)	81/57 (54.3/45.7)	171/144 (58.7/41.3)	0.385
Age (years)	70.32±8.26	73.44±8.98	0.001
Height (cm)	160 (155-169)	163 (158-170)	0.071
Weight (kg)	80 (72-90)	80 (74-92)	0.291
BMI	30.89 (26.67-35.18)	30.22 (27.64-34.05)	0.793
SBP (mmHg)	140 (125-140)	135 (120-145)	0.423
DBP (mmHg)	80 (70-80)	80 (70-85)	0.041
HR (bpm)	70 (63-79)	86 (74-98)	<0.001
HT n (%)	103 (74.6)	249 (79)	0.299
DM n (%)	36 (26.1)	131 (41.6)	0.002
CAD n (%)	26 (18.8)	75 (23.8)	0.242
PAD n (%)	9 (6.5)	18 (5.7)	0.738
Smoking status n (%)	14 (10.1)	18 (5.7)	0.090
Alcohol drinking n (%)	15 (10.9)	36 (11.4)	0.862
COPD n (%)	8 (5.8)	21 (6.7)	0.728
Asthma n (%)	6 (4.3)	10 (3.2)	0.583
Symptomatic status			
• Asymptomatic • Symptomatic	58 (42) 80 (58)	221 (70.2) 94 (29.8)	<0.001
Ablation treatment			
• Received • Did not receive	26 (18.8) 112 (81.2)	32 (10.2) 283 (89.8)	0.011
NOAC n (%)	117 (84.8)	266 (84.4)	0.927
Warfarin n (%)	21 (15.2)	49 (15.6)	0.927
ACEI/ARB n (%)	86 (62.3)	190 (60.3)	0.688
BB n (%)	78 (56.5)	195 (61.9)	0.281
DHP-CCB n (%)	38 (28.4)	91 (30.4)	0.662
Non-DHP-CCB n (%)	11 (8)	30 (9.5)	0.596
Statin n (%)	34 (24.6)	54 (17.1)	0.063

*Non-parametric continuous data are presented as median (1st quartile to 3rd quartile). ACEI: angiotensin-converting enzyme inhibitors; AF: atrial fibrillation; ARB: angiotensin receptor blockers; BB: beta blockers; BMI: body mass index; CAD: coronary artery disease; COPD: chronic obstructive pulmonary disease; DBP: diastolic blood pressure; DHP-CCB: dihydropyridine calcium channel blockers; DM: diabetes mellitus; F: female; HR: heart rate; HT: hypertension; M: male; NOAC: non-vitamin K antagonist oral anticoagulant; PAD: peripheral artery disease; SBP: systolic blood pressure.*

Conventional inflammatory markers such as neutrophil count, NLR, PLR, SII, CRP, and red blood cell distribution width (RDW) were significantly higher in patients with permanent AF than in patients with paroxysmal AF. On the contrary, the mean lymphocyte count of patients with permanent AF was significantly lower than that of patients with paroxysmal AF. However, WBC, hemoglobin, and platelet count did not differ significantly between groups, as shown in Table 2. Likewise, kidney, liver, and thyroid function tests, as well as lipid panel and fasting blood glucose results did not differ significantly between the two groups. In contrast, hemoglobin A1C was significantly higher in patients with permanent AF than in patients with paroxysmal AF. Regarding the echocardiographic data, the ejection fraction of both groups was similar, but the left atrial diameter of patients with permanent AF was significantly larger than that of patients with paroxysmal AF.

**Table 2:** – Laboratory and echocardiographic characteristics of the study population

Variables	Paroxysmal AF 138	Permanent AF 315	p
WBC (×10^3^/µL)	7.04±1.78	7.28±1.91	0.218
Hemoglobin (g/dL)	13.26±1.69	12.93±1.84	0.070
Neutrophil (×10^3^/µL)	3.875 (2.958-5.000)	4.400 (3.480-5.770)	<0.001
Lymphocyte (×10^3^/µL)	2.15±0.67	1.82±0.7	<0.001
Platelet (×10^3^/µL)	229.5 (191.0-288.5)	229 (198-272)	0.743
PLR	109.75 (90.46-139.90)	133.94 (98.15-180.95)	<0.001
NLR	1.86 (1.36-2.57)	2.39 (1.85-3.47)	<0.001
SII	444.11 (309.25-601.12)	562.50 (386.41-897.66)	<0.001
CRP (mg/L)	2.42 (1.58-5.10)	4.60 (2.20-11.10)	<0.001
RDW (%)	14.5 (13.7-15.3)	15.0 (14.2-16.3)	<0.001
Creatinine (mg/dL)	0.84±0.15	0.86±0.17	0.246
GFR (ml/minute)	83.27 (68.44-99.45)	75.76 (65.95-93.54)	0.051
ALT (U/L)	15 (11-21)	15 (11-20)	0.989
AST (U/L)	18 (16-23)	19 (15-23)	0.959
TSH (ng/dL)	2.19 (1.08-3.15)	2.2 (1.41-3.15)	0.278
T3 (ng/dL)	3.8 (2.7-4.6)	4 (3.07-4.60)	0.171
T4 (ng/dL)	16.45 (14.70-18.20)	16.20 14.40-18.20)	0.565
FBG (mg/dL)	109 (98-131)	114 (98-145)	0.111
HBA1C (%)	5.8 (5.5-6.3)	5.9 (5.54-6.90)	0.047
TC (mg/dL)	188.55±43.04	180.60±41.46	0.065
LDL-C (mg/dL)	109 (81-134)	107 (82-128)	0.344
HDL-C (mg/dL)	47 (39-56)	46 (39-53)	0.080
TG (mg/dL)	127 (99-183)	124 (91-165)	0.173
EF (%)	65 (60-65)	65 (60-65)	0.503
LAD (cm)	38.6 (38.0-41.0)	46 (43-49)	<0.001

*Non-parametric continuous data are presented as median (first quartile to third quartile). AF: atrial fibrillation; ALT: alanine aminotransferase; AST: aspartate aminotransferase; CRP: C-reactive protein; EF: ejection fraction; FBG: fasting blood glucose; HBA1C: hemoglobin A1C; HDL-C: high-density lipoprotein cholesterol; GFR: glomerular filtration rate; LAD: left atrial diameter; LDL-C: low-density lipoprotein cholesterol; NLR: neutrophil-to-lymphocyte ratio; PLR: platelet-to-lymphocyte ratio; RDW: red blood cell distribution width; SII: systemic immune-inflammation index; T3: triiodothyronine; T4: tetraiodothyronine; TC: total cholesterol; TSH: thyroid-stimulating hormone; TG: triglyceride; WBC: white blood cell.*

A binary logistic regression analysis was performed to identify the independent variables that influence AF permanence. For this purpose, the effect of age, sex, body mass index (BMI), hypertension, coronary artery disease, DM, chronic obstructive pulmonary disease, WBC, RDW, CRP, SII, and left atrial anterior-posterior diameter on the pattern of AF were examined. The full model with all aforementioned variables was statistically significant (X^2^ [12, n = 453] = 324.20, p < 0.001), indicating that the model was able to distinguish the pattern of AF as paroxysmal or permanent. Our model fit the data well (good fit) with a Hosmer and Lemeshow test significance value of p = 0.947. In addition, the model had a high percentage (88.5%) of prediction accuracy. Moreover, 51.1% (Cox and Snell R square) to 72.2% (Nagelkerke R square) of the variance in the AF pattern could be explained by the model. As seen in Table 3, age, DM, WBC, RDW, CRP and SII variables were significantly associated with AF pattern. Considering the age, the odds of having permanent AF increased by 1.046 times with each year increase in age. Similarly, individuals with DM had 2.2 times greater odds of having permanent AF than patients without DM. RDW had an odds ratio (OR) of 1.31, meaning that the odds of having permanent AF increased by 1.31 times per unit increase in RDW. Likewise, a unit increase in CRP increased the odds of having permanent AF by 1.31 times. SII was another significant predictor of permanent AF (OR: 1.002, p = 0.001). This means that for each unit increase in SII, the odds of having permanent AF changed by a factor of 1.002. Left atrial anterior-posterior diameter was detected as the most powerfully associated with permanent AF in this model, with an OR of 2.04, indicating that each centimeter increase in left atrial diameter increased the odds of having permanent AF by 2.04 times, as shown in Table 3.

**Table 3:** – Binary logistic regression analysis for the likelihood of atrial fibrillation pattern

Variables	B	SE	Wald	df	p	OR	95% CI for OR	
							Lower	Upper
Age	0.045	0.022	4.288	1	0.038	1.046	1.002	1.092
Sex (female)	−0.471	0.372	1.608	1	0.205	0.624	0.301	1.293
BMI	−0.012	0.033	0.134	1	0.714	0.988	0.926	1.054
HT (present)	−0.304	0.412	0.545	1	0.460	0.738	0.329	1.654
CAD (present)	−0.085	0.441	0.037	1	0.847	0.919	0.387	2.179
DM (present)	0.789	0.387	4.153	1	0.042	2.201	1.031	4.698
COPD (present)	0.350	0.751	0.218	1	0.641	1.420	0.326	6.181
WBC	−0.084	0.110	0.581	1	0.446	0.919	0.741	1.141
RDW	0.270	0.119	5.137	1	0.023	1.310	1.037	1.654
CRP	0.108	0.042	6.595	1	0.010	1.114	1.026	1.210
SII	0.002	0.001	10.414	1	0.001	1.002	1.001	1.003
LAD	0.713	0.076	87.486	1	<0.001	2.040	1.757	2.369
Constant	−36.644	4.555	64.726	1	<0.001	0.000		

*BMI: body mass index; CAD: coronary artery disease; CI: confidence interval; COPD: chronic obstructive pulmonary disease; CRP: C-reactive protein; df: degrees of freedom; DM: diabetes mellitus; HT: hypertension; LAD: left atrial diameter; OR: odds ratio; RDW: red blood cell distribution width; SE: standard error; SII: systemic immune inflammation index; WBC: white blood cell.*

### Patients with diabetes

Supplementary Table  describes some features of patients with diabetes. A total of 167 (36.9%) out of 453 patients had diabetes. The median BMI of patients with DM was 30.81 (27.64 to 34.68), and they had diabetes for a median of 10 (6 to 13) years. Of these 167 patients, 147 (88%) were on oral antidiabetic drugs, and 40 (24%) were on insulin therapy. The majority (n = 131, 78.4%) of these patients had permanent AF. RDW as an inflammatory marker was significantly higher in patients with DM in comparison to those without DM.

### Ablation treatment

In the present sample, 58 (12.8%) patients underwent ablation. Patients who underwent ablation were significantly younger, more often male, and had lower BMI. However, the frequency of smoking was higher in patients who underwent ablation (Supplementary Table 2).

### Symptomatic/asymptomatic status of patients

While most patients with paroxysmal AF were significantly symptomatic (80/138), most patients with permanent AF were asymptomatic (221/315). BMI, the frequency of DM, coronary artery disease, and chronic obstructive pulmonary disease were significantly higher in symptomatic patients. In the asymptomatic group, the use of beta-blocking agents was significantly higher (Supplementary Table 3).

## Discussion

The objective of this study was to investigate the relationship between inflammation and AF burden. We found that patients with permanent AF had a greater inflammation burden than patients with paroxysmal AF. This was evidenced by significantly higher levels of neutrophils, PLR, NLR, SII, CRP, and RDW in patients with permanent AF. Additionally, age, diabetes, RDW, CRP, SII (central illustration), and left atrial anterior-posterior diameter were independently associated with the permanent AF pattern, which has the qualitatively highest AF burden.

Inflammation is thought to play a pivotal role in the pathogenesis of AF. Inflammatory markers have been shown to be elevated in patients with AF, and the inflammation burden has been shown to be associated with AF burden and prognosis. The exact mechanisms by which inflammation contributes to AF are not fully understood, but they may involve electrical and structural remodeling of the atria.^1,5^

The prevalence of AF increases with age in both sexes, doubling every 10 years and reaching up to 20% in people over 80 years old.^1,9,10^ Older age is also an independent predictor of permanent AF, as shown by a 30-year follow-up study that reported a cumulative probability of 29% for non-permanent AF to progress to permanent AF.^1,11^ Our findings are consistent with previous studies that demonstrated a positive association between age and AF progression.^10-12^ The possible mechanisms underlying this association include the higher incidence of comorbidities, inflammation, and atrial fibrosis in the elderly population.^13^

DM is a well-established risk factor for the development of AF, with a 34% higher risk compared to individuals without DM.^4,14^ However, the role of diabetes in the maintenance and progression of AF is not fully understood, and the results of previous studies are conflicting.^1,15,16^ In this study, permanent AF was significantly higher in patients with DM than in those without DM. In addition, RDW as an inflammatory marker was significantly higher in patients with DM in comparison to those without DM. In our study, we found a positive and independent association between diabetes and permanent AF. Despite different design, population characteristics, and smaller sample sizes compared to previous studies, similar results were obtained between DM and AF in the present analysis. This relationship can be explained by the high inflammation burden in DM.

Left atrial enlargement is a consequence of increased atrial pressure and volume, as well as left ventricular diastolic dysfunction, which lead to electrical and structural remodeling of the left atrium and predispose to AF initiation and progression.^1,4^ Conversely, AF causes further atrial dilation, creating a vicious cycle. A recent study by Menichelli et al.^17^ reported a significantly higher median left atrial diameter in patients with permanent AF than in those with paroxysmal AF (≥ 44 mm, 59.5% versus 37.5% respectively, p < 0.001). Moreover, permanent AF and left atrial enlargement are associated with a higher risk of ischemic stroke and systemic embolism.^4,17,18^Furthermore, a recent study found a significantly higher degree of left atrial remodeling in terms of more enlarged size and more impaired function (stiff left atrium) in patients with permanent as compared with paroxysmal AF, reflecting different stages of the disease.^19^ In this context, our analysis showed that left atrial anterior-posterior diameter (a simplified measure of left atrial size) was associated with permanent AF.

Inflammation is considered to be both a potential trigger and a perpetuating factor in the pathophysiology of AF.^5^ Several case-control studies have shown higher levels of inflammatory markers in patients with AF compared to those without AF.^5,20-22^ Furthermore, the inflammatory burden and atrial fibrosis are positively correlated with AF burden.^5,23^ Smit et al.^24^ reported that inflammatory markers were associated with the development of permanent AF. In a large population-based study, Aviles et al. reported that increased CRP was associated with the presence of AF and predicted future AF.^6^ Similarly, our study found that permanent AF was associated with higher CRP levels than paroxysmal AF, suggesting that CRP levels may reflect AF burden.^25^ RDW is another important indicator of inflammation and has been linked to AF occurrence, recurrence, permanence, and AF-related adverse events.^26^ Additionally, RDW was shown to be an independent predictor of postoperative AF after coronary artery bypass grafting.^27^ Furthermore, elevated RDW has been suggested as an independent predictor of long-term adverse clinical outcomes.^26,28^ Wan et al. reported that individuals with high RDW levels exhibited a significantly higher prevalence of persistent and permanent AF.^28^ Furthermore, RDW has been independently associated with AF progression from paroxysmal to permanent.^29^ Our results support previous literature by observing an independent association between RDW values and permanent AF. In summary, our findings regarding inflammation markers such as RDW, CRP, and SII show that they are associated with the burden and permanence of the AF.

SII is a novel inflammatory marker that combines platelet, neutrophil, and lymphocyte counts, and may reflect the immune and inflammatory status more accurately than any of these cells alone. Elevated SII has been shown to be significantly associated with various cardiovascular events and outcomes.^9,30-32^Moreover, SII has been proposed as a predictor of AF development after coronary artery bypass grafting,^33^ AF recurrence after cryo-maze procedure with mitral valve surgery,^31,34^ and after successful direct current cardioversion.^35^The present study attempted to explore the association between AF burden, which is theoretically and qualitatively highest in permanent AF and lowest in paroxysmal AF, and SII, in a particular method and with a more simplified view.

Considering that inflammation is closely related to the development, burden, and progression of AF, possible preventive measures may prevent all these steps.^6,36^ Additionally, agents targeting inflammatory biomarkers have recently begun to be investigated as potential drugs in the treatment of AF.^6,36,37^ Furthermore, colchicine treatment after pulmonary vein isolation for paroxysmal AF is associated with lower AF recurrence rate.^34^ It indicates that reduction of these pro-inflammatory markers could guide the choice of the best patient profiles for response to clinical treatment or catheter intervention.

Catheter ablation is increasingly used for rhythm control in the treatment of AF.^1^ It is known that demographic characteristics have a significant impact on the treatment response and outcomes of catheter ablation.^1,38^ In a study by Kummer et al., patients who underwent catheter ablation were significantly younger, more often male, more often White, and more often privately insured, with higher household incomes and lower rates of medical comorbidity.^39^ In the present sample all patients were insured through government-sponsored insurance programs and 12.8% (58/453) of patients underwent ablation. Patients who underwent ablation more often had paroxysmal AF, were significantly younger men, and had lower BMI.

Although AF is often asymptomatic, it can be disabling.^1^ AF type and the presence of comorbidities are effective factors in whether AF patients are symptomatic or not.^1,40^ Permanent AF has been reported to be 3 times more common in asymptomatic patients than in symptomatic patients.^40^ Additionally, male sex, older age, previous myocardial infarction and limited physical activity have been shown to be significantly associated with asymptomatic AF.^40^ Consistent with previous literature, the frequency of paroxysmal AF in symptomatic patients and the frequency of permanent AF in asymptomatic patients were significantly higher. Likewise, comorbidities were significantly higher in the symptomatic group.

### Limitations

First, AF burden is a complex concept that cannot be measured precisely. In the current analysis, AF burden was roughly estimated from AF patterns, being qualitatively lowest and highest in paroxysmal and permanent AF, respectively. Second, this was a single-center cross-sectional study; thus, it cannot prove causality. Long-term prospective follow-up studies are needed to validate our findings and investigate the underlying mechanisms of inflammation in AF. Third, the currently used clinical AF classifications poorly reflect the temporal persistence of AF. In addition, patients classified in the same clinical AF class may be inherently heterogeneous in terms of temporal AF persistence.^41^ We sought to explore the relationship between SII and two distinctly separate stages of AF, paroxysmal and permanent (theoretically those with the lowest and highest AF burden). Therefore, the study did not include a control group without AF but with the same demographic characteristics or a group with the persistent and long-standing persistent forms of AF. However, it would be more valuable to reveal the gradual relationship between increasing AF burden (presumably according to the temporality of AF from paroxysmal to persistent, long-standing persistent, and permanent) and SII. Therefore, well-designed prospective studies that will make sense of our research are needed. Fourth, our study population was relatively small and consisted of elderly Caucasian patients; therefore, the generalizability of our findings to other populations is uncertain. Fifth, we used only left atrial anteroposterior diameter, a simple parameter that does not truly reflect left atrial size, particularly in case of asymmetric left atrium. Therefore, studies evaluating left atrial volume to determine left atrial size will contribute to our analysis.

## Conclusion

In addition to left atrial enlargement, inflammation burden as represented by SII, NLR, CRP, and RDW is independently associated with AF burden. Our study provides important insights into the relationship between inflammation and AF burden. However, further research is needed to validate our findings and investigate the potential role of anti-inflammatory therapies in the prevention or treatment of AF.
